# A new pilot shared method for saving bandwidth cost of OFDM

**DOI:** 10.1038/s41598-024-55153-y

**Published:** 2024-02-24

**Authors:** Shahid Ali, Dongsheng Zheng, Bingli Jiao

**Affiliations:** School of Electronics, Peking University, Beijing, 100871 P. R. China

**Keywords:** Frequency selective channel, Shared pilot design, Spectral efficiency, Engineering, Electrical and electronic engineering

## Abstract

The orthogonal frequency division multiplexing (OFDM) system applies coherent demodulation to achieve high spectral efficiency at a bandwidth cost by the pilot tones. Considering the statistical property of the down-link channels to the users, it can be found that there is an opportunity to reduce the pilot number in the conventional designs while maintaining the same signal demodulation performances. The design philosophy involves utilizing the difference of the channel coherent bandwidths (CCBs) by allocating data to appropriate positions upon the fact that different CCBs can tolerate different minimized pilot spacing. The proposed design allows each user’s equipment’s data not to exceed its CCB with the sparser pilots. The theoretical analysis is carried out based on the concept of channel frequency response using linear interpolation with channel estimation employing the least squares (LS) method. The gain of the proposed method is demonstrated in terms of the ergodic capacities and confirmed by the simulations.

## Introduction

Orthogonal frequency division multiplexing (OFDM) techniques are extensively employed in communication systems, such as LTE and 5G systems^1^, due to the inherent nature of dealing with the frequency-selective fading channels^2^. The transmission of pilots is crucial to facilitate coherent signal detection^3,4^ at the receivers, of which the communication channels can be statistically different depending on their channel delay spreads, e.g., in urban or rural environment^5,6^.

As has been proven, the optimum pilot design for an OFDM system is the arrangement of equally spaced constant-power pilots in both the time and frequency domains^7,8^. Although the pilot designs appear to be mature in many existing systems, there are still chances for further increasing spectral efficiency, especially when 6G wireless is considered for the possible applications involving territorial, air, sea, and space communications^9^. In light of these advancements, one base station (BS) can transmit signals to the user-equipments (UEs) having significantly different CCBs, of which the larger CCBs are considered as the advantage to increase the spectral efficiency in this paper.

To clearly explain the new design philosophy, our research focuses on the frequency domain with the comb-type pilot structure and channel estimation based on calculations of the channel frequency response (CFR)^10,11^. Our work begins with channel estimation using the LS method, followed by calculations of the CFR^12,13^, and then estimations of the ergodic capacity.

The pilot spacing is the major issue to be considered for its influence on the spectral efficiency of the system in connection to the accuracy of the CFRs of UEs over the frequency-selective fading channels^14^.

**Figure 1:**
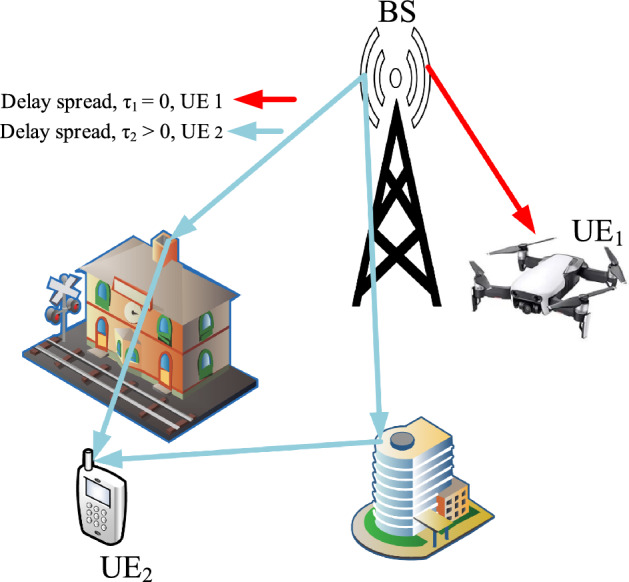
Different delay spread users in various channel situation scenarios.

To illustrate the above argument, downlink channels of one BS are considered for its signal transmission to the two UEs, as an example, depicted in Fig. 1, where the scenario includes two extreme cases of CCBs: one UE is a drone and the other is a conventional ground-based UE. The former can receive the signal through a single-path channel, while the latter is the multipath channel due to the local signal reflections. It is well-known that the CCB of a single-path channel is infinitely large and, however, that of the multipath channel is much smaller, depending on the delay spreads^15^. The CCB is inversely proportional to the delay spread^16^.

A weakness of the conventional pilot design lies in its conservative approach, which takes the smallest CCB of all UEs to determine the pilot spacing^17^. This approach aims to guarantee the performance for the worst-case scenario of the UEs, despite other UEs potentially having better channel situations with larger CCBs. Consequently, the pilot arrangement becomes overly condensed, leading to unnecessarily high bandwidth costs.

Motivated by the potential benefits associated with larger CCBs, we propose a novel method to increase the pilot spacing in the comb-type structure by allowing the UEs of different CCBs to share pilots in different positions. Thus, the proposed design is referred to as the pilot-shared (PS) method.

To facilitate the PS method in system operation, the BS needs to schedule the data of each UE according to its CCB onto the subcarriers with appropriate distances concerning the pilots. Additionally, it needs to inform each UE of its data locations. It is noted that the PS method introduces minimal complexity when applied to a time division duplex (TDD) system because all CCBs can be obtained directly at the BS due to channel reciprocity. Furthermore, the bandwidth consumption attributed to signaling for informing the data locations is insignificant. This is because the CCB of each UE primarily depends on the channel delay spread environment, which varies at a much slower rate compared to the communication data frame rate.

The remainder of this paper is organized as follows. “Preliminaries” explains the basics of OFDM systems and the channel estimation method as preliminaries. “New pilot shared design” presents the PS method and the theoretical derivations, including the calculation of ergodic capacity. “Performance evaluation” evaluates the spectral efficiency to show the advantage of the PS method in terms of ergodic capacities and “Conclusion” concludes the paper.

## Preliminaries

To provide a basis for explaining the PS method, we refer back to the concept of channel frequency response^18^ along with the utilization of the interpolation technique^11,19^. The LS method is employed for channel estimations in this context.

### Channel estimation in OFDM transmission

**Figure 2:**
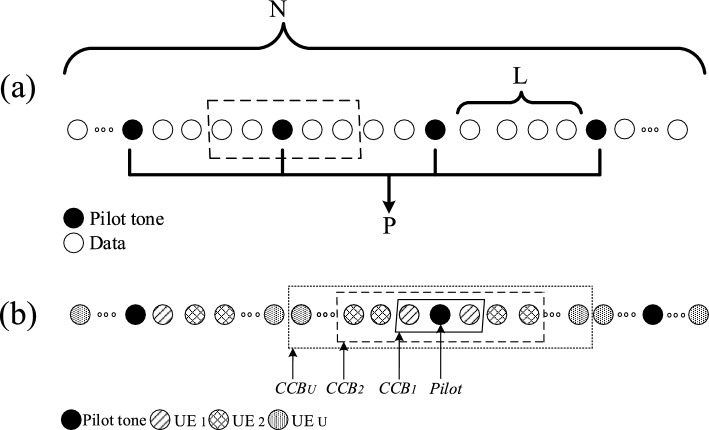
An illustration of comb-type pilot structure.

Let us depict the conventional comb-type pilot design of OFDM in one dimension in Fig. 2a, where the dashed rectangle illustrates the pilot spacing in the conventional design. The solid circles represent the pilot symbols, while the white circles denote the UEs’ data.

The transmitted signal of the OFDM is expressed in vector form by ${\textbf{X}}_N = \left[ X_1, X_2,\ldots , X_n, \ldots , X_{N} \right] ^{\textrm{T}}$, where *N* is the total number of subcarriers with $n = 1, 2, \ldots , N$. For showing the performance of the pilots, ${\textbf{X}}_P= \left[ X_1, X_{L+1},\ldots , X_{pL+1},\ldots , X_{PL+1}\right] ^{\textrm{T}}$ is used to express the pilot vector in the following derivations, where $p = 1, 2,..., P+1$ is the index of the pilot subcarrier and *L* is the pilot spacing in terms of subcarriers with the assumption of $L=N/P$ in the design. The transmission of the pilots can be expressed by

1 $$\begin{aligned} {\textbf{Y}}_P={\textbf{H}}_P{\textbf{X}}_P+{\textbf{W}}_P, \end{aligned}$$

where ${{\textbf{Y}}}_P = [ Y_1, Y_{L+1},\ldots , Y_{pL+1},\ldots , Y_{PL+1}] ^{\text{T}}$ is the received signal at the receiver, ${{\textbf{X}}}_P = [ X_1, X_{L+1},\ldots , X_{pL+1},\ldots , X_{PL+1}] ^{\text{T}}$ is the transmit signals, ${\textbf{H}}_P$ is the channel matrix in form of diagonal matrix with the element ${H}_{pL}$ as the channel gain of $X_{pL}$ and the noise matrix ${\textbf{W}}_P= \left[ W_1, W_{L+1},\ldots , W_{pL+1},\ldots , W_{PL+1}\right] ^{\textrm{T}}\nonumber$ is the Gaussian noise, respectively.

The elements ${Y}_{pL}$, ${X}_{pL}$ and ${H}_{pL}$ represent the received, transmitted pilot and the channel gain on the *p*-th subcarrier for $p =1,2,..., P+1$, respectively. It is noted that index *p* represents the order of the pilots and *L* the pilot interval in terms of subcarrier, and the channel gain is distributed in one dimension manner because of its diagonal form of the channel matrix. The noise $W_{pL}$ is assumed to obey the identical independent Gaussian distribution. The frequency coherency among ${H}_{pL}$ for ${pL} = 1,2,...N$ will be considered in frequency correlation function $R_f$ in the next sections.

With the received pilot tones ${\textbf{Y}}_P$, the estimated channel coefficients ${\hat{\mathbf{H}}}_P$ at pilot tones can be calculated by different metrics^20^, e.g., the least-square (LS) channel estimation^21^, and the minimum mean square error (MMSE) channel estimation^22^. The LS estimation method has been widely adopted for its simplicity. Specifically, the estimated channel coefficients for each pilot tone can be represented as

2 $$\begin{aligned} {\hat{H}}_{pL} = \dfrac{{Y}_{pL}}{{ X}_{pL}}={ H}_{pL}+\dfrac{{ W}_{pL}}{{ X}_{pL}}, \end{aligned}$$

and mean-square error (MSE) of the LS channel estimation method is

3 $$\begin{aligned} \varepsilon _{p}=\sigma _W^2/\sigma _X^2\triangleq 1/\rho , \end{aligned}$$

where, $\sigma _W^2$ is the AWGN noise power, and $\rho$ is the pilot SNR.

### Linear interpolation

Once the channel coefficients at the pilot tones are obtained in Eq. (2), interpolation techniques in the time domain and/or frequency domain are necessary to obtain the estimated channel coefficients at the data subcarriers. For simplicity of the problem formulation, the linear interpolation method is adopted in this work. However, we remark that this work can be extended to other interpolation techniques, e.g., second-order polynomial interpolations and cubic spline interpolations^23,24^. In terms of linear interpolation, the estimated channel coefficients at the data subcarriers are formulated as^25^

4 $$\begin{aligned} {\hat{H}}_{pL+l}={\hat{H}}_{pL}+\frac{l}{L}\left[ {{\hat{H}}_{(p+1)L}-{\hat{H}}_{pL}}\right] , \end{aligned}$$

with $l=1,2,\ldots ,L-1$, ${\hat{H}}_{pL}$ and ${\hat{H}}_{(p+1)L}$ denote the estimated channel coefficients of the *p*-th and the $(p+1)$-th pilot tone, respectively.

5 $$\begin{aligned} \varepsilon _{pL+l}&= \mathbb {E}\left\{ \left\| {H}_{pL+l}-{\hat{H}}_{pL+l}\right\| ^2\right\} =\mathbb {E} \left\{ \left\| {H}_{pL+l}-\frac{L-l}{L}{\hat{H}}_{pL}-\frac{l}{L}{\hat{H}}_{(p+1)L}\right\| ^2\right\} \nonumber \\&=\mathbb {E}\left\{ \left\| {H}_{pL+l}-\frac{L-l}{L}{H}_{pL}-\frac{l}{L}{H}_{(p+1)L} -\frac{L-l}{L}\dfrac{W[pL]}{X[pL]}-\dfrac{l}{L}\dfrac{W[(p+1)L]}{X[(p+1)L]}\right\| ^2\right\} \nonumber \\&=\mathbb {E}\left\{ \left\| {H}_{pL+l}\right\| ^2\right\} +\frac{(L-l)^2}{L^2}\mathbb {E} \left\{ \left\| {H}_{pL}\right\| ^2\right\} +\frac{l^2}{L^2}\mathbb {E} \left\{ \left\| {H}_{(p+1)L}\right\| ^2\right\} +\frac{(L-l)l}{L^2}\mathbb {E} \left\{ {H}_{pL}{H}^*_{(p+1)L}+{H}_{(p+1)L}{H}^*_{pL}\right\} \nonumber \\&\quad -\frac{L-l}{L}\mathbb {E}\left\{ {H}_{pL}{H}^*_{pL+l}+{H}_{pL+l}{H}^*_{pL}\right\} -\frac{l}{L}\mathbb {E} \left\{ {H}_{pL+l}{H}^*_{(p+1)L}+{H}_{(p+1)L}{H}^*_{pL+l}\right\} +\frac{(L-l)^2+l^2}{\rho L^2} \nonumber \\&=\frac{(L-l)^2+L^2+l^2}{L^2}R_f(0)+\frac{(L-l)^2+l^2}{\rho L^2}+\frac{(L-l)l}{L^2}\left[ R_f(L)+R_f(-L)\right] \nonumber \\&\quad -\frac{L-l}{L}\left[ R_f(l)+R_f(-l)\right] -\frac{l}{L}\left[ R_f(L-l)+R_f(l-L)\right] \end{aligned}$$

Then, the mean square error (MSE) of the estimation at the $(pL+l)$-th data subcarrier $\varepsilon _{pL+l}$ is given in Eq. (5), which is shown at the top of the next page.

In Eq. (5), the frequency correlation function $R_f(l)$ is defined as

6 $$\begin{aligned} R_f(l)\triangleq \mathbb {E}\left\{ {H}_{pL}{H}^*_{pL+l}\right\} , \end{aligned}$$

where $\mathbb {E}\left\{ \cdot \right\}$ is the expectation (mean) operator. Observe from Eq. (5) that the estimation error is determined by the relative position of the pilot tones. Therefore, we express the estimation MSE $\varepsilon _{pL+l}$ as $\varepsilon _{l}$ thereafter.

### Imperfect CSI

By elaborating the MSE in Eq. (3), the channel coefficient at the *l*-th subcarrier *H*[*l*] can be expressed as^26,27^,

7 $$\begin{aligned} H[l]={\hat{H}}[l]+\tilde{H}[l], \end{aligned}$$

where ${\hat{H}}[l]$ is the estimated channel coefficient, and $\tilde{H}[l]$ is the estimation error, with $l = 1, 2, \ldots , L-1$ . Furthermore, it is assumed that *H*[*l*] and $\tilde{H}[l]$ follow an independent and identically distributed (i.i.d.) zero-mean complex Gaussian distribution with variances of 1 and $\varepsilon _{l}$, respectively. Then, the received signal is formulated as

8 $$\begin{aligned} Y[l]&=H[l]X[l]+W[l]\nonumber \\&={\hat{H}}[l]X[l]+\tilde{H}[l]X[l]+W[l]. \end{aligned}$$

The received signal to interference-plus noise ratio (SINR) can be obtained by

9 $$\begin{aligned} \textrm{SINR}[l] = \dfrac{|{\hat{H}}[l]|^2|X[l]|^2}{|\tilde{H}[l]|^2|X[l]|^2+\sigma _W^2}=\dfrac{\rho |{\hat{H}}[l]|^2}{1+\rho |\tilde{H}[l]|^2}, \end{aligned}$$

where $\rho = |X[l]|^2/\sigma _W^2$ is the SNR, $|\cdot |$ is the modulus operator and ${\hat{H}}[l]$ is the estimated channel coefficient obeying complex Gaussian distribution of zero mean and the variance of $1-\varepsilon _{l}$.

## New pilot shared design

**Figure 3:**
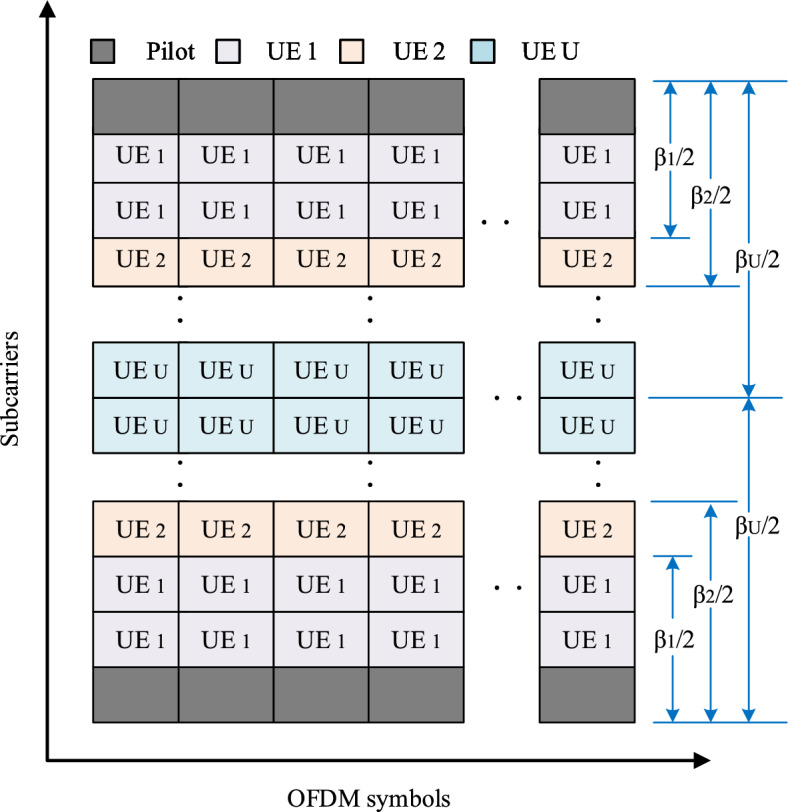
A sub-block with PS method to different CCB users’ equipments.

This section introduces the PS method by considering the difference of the CCBs^28^ over the downlink channels as shown in Fig. 2b, where the pilot spacing has been increased.

For illustrating the PS method clearly, we assume that one BS transmits the signals to *U* UEs, of which the channel delay spreads are uniformly ranged in the region of $[\bar{\tau }_1, \bar{\tau }_U]$without overlap onto each other, where *U* is the total number of users.

By arranging the delay spreads in terms of $\bar{\tau }_u$ with $\bar{\tau }_1> \bar{\tau }_2>,...>\bar{\tau }_u>,....,>\bar{\tau }_U$, the corresponding CCB can be calculated by

10 $$\begin{aligned} {\beta }_u \approx \frac{1}{\bar{\tau }_u}, \end{aligned}$$

with ${\beta }_1<{\beta }_2<\cdots<{\beta }_u<\cdots <{\beta }_U$, where $\bar{\tau }_u$ and $\beta _u$ are the delay spread and the coherent bandwidth of the UE_u_ for $u = 1,2,\ldots , U$, respectively.

To complete the model of the PS method, the following operations are added to the conventional system. (1) The users’ data are set onto the data subcarriers in such a way that the smaller the CCB is, the closer position the corresponding user’s data are allocated. Consequently, the UE data of the smallest CCB is arranged in the region adjacent to the pilot symbols and those having the largest CCB in the middle region between two pilot symbols. (2) The data allocations are successfully transmitted to all users so that each UE can identify its data in the frequency domain in performing its coherent demodulation.

Then, by adopting the exponential-decay power delay spread profile to the channel model of this work, the delay spread of a UE can be characterized by^29^

11 $$\begin{aligned} S_u(\tau ) = {\left\{ \begin{array}{ll} \dfrac{1}{\bar{\tau }_u}\exp {\left( -\dfrac{\tau }{\bar{\tau }_u}\right) },\quad &{}\textrm{for} \ \tau \ge 0\\ 0,\quad &{}\textrm{for} \ \tau < 0, \end{array}\right. } \end{aligned}$$

where $\bar{\tau }_u = \int _{-\infty }^{\infty } S_u(\tau ) \tau \textrm{d} \tau$ denotes the delay spread of the *u*-th UE. In considering the expression of CCBs, the delay spreads of the UEs are arranged in a manner of $\bar{\tau }_1> \bar{\tau }_2> \cdots>\bar{\tau }_u>\cdots >\bar{\tau }_U$.

According to^30^, the correlation function $R_f(l)$ can be formulated as

12 $$\begin{aligned} R_f(l)&= \int _{-\infty }^{\infty } S(\tau ) \exp {\left( -j 2 \pi \tau l\Delta f\right) } \textrm{d} \tau \nonumber \\ {}&=\int _{0}^{\infty } \dfrac{1}{\bar{\tau }_u} \exp {\left[ -\left( \dfrac{1}{\bar{\tau }_u}+j 2 \pi l\Delta f\right) \tau \right] } \textrm{d} \tau \nonumber \\&=\frac{1}{1+j2\pi l\Delta f \bar{\tau }_u}, \end{aligned}$$

where $\Delta f$ denotes the subcarrier bandwidth.

Substituting Eq. (12) into Eq. (5), we can obtain the MSE of estimation at the *l*-th data subcarrier for the *u*-th UE as

13 $$\begin{aligned} \varepsilon _{u,l}&= \frac{(L-l)^2+L^2+l^2}{L^2}+\frac{(L-l)^2+l^2}{\rho L^2}\nonumber \\&\quad +\frac{2(L-l)l}{L^2\left[ 1+4\pi ^2L^2(\Delta f)^2\bar{\tau }_u^2\right] }-\frac{2(L-l)}{L\left[ 1+4\pi ^2l^2(\Delta f)^2\bar{\tau }_u^2\right] } \nonumber \\&\quad -\frac{2l}{L\left[ 1+4\pi ^2(L-l)^2(\Delta f)^2\bar{\tau }_u^2\right] }, \end{aligned}$$

which indicates that the MSE $\varepsilon _{u,l}$ of the *l*-th data subcarrier is the same as that MSE $\varepsilon _{u,L-l}$ of the $(L-l)$-th data subcarrier, that is, $\varepsilon _{u,l}=\varepsilon _{u,L-l}$, with $l = 1,2,\ldots , L-1$. Furthermore, it is found that the estimation MSE at the *l*-th data subcarrier increases as the delay spread of the UE is exacerbated. Based on these observations, our PS scheme is stated as follows.

**Figure 4:**
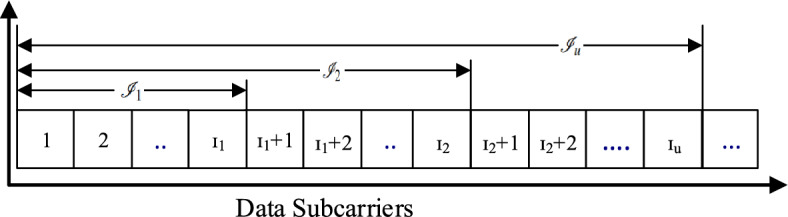
Data subcarriers allocation scheme.

Figure 3 shows the data format in terms of the subcarriers, where subcarriers in closer proximity to the pilot tones are allocated to the UE with the smallest coherence bandwidth ${\beta }_u$. In addition, we distribute the *l*-th and $(L-l)$-th data subcarriers to the same UE. The smaller indices of the data subcarriers allocated to UE *u* are denoted by ${\mathscr{I}}_u$, i.e., ${\mathscr{I}}_1=\left\{ 1,2,\ldots ,I_1\right\}$, and ${\mathscr{I}}_2=\left\{ I_1+1,I_1+2,\ldots ,I_2\right\}$, with $I_u\in \mathbb {Z}^{+}$ and $I_u\le L/2$. It is noted that ${\mathscr{I}}_u$ is an empty set, that is, ${\mathscr{I}}_u=\emptyset$, when $I_u=I_{u-1}$ holds, and $I_0$ is defined as $I_0=0$, Fig. 4, clarified the data subcarriers allocation with symbols.

Referring to Eq. (9), the SINR of the individual subcarrier is given by

14 $$\begin{aligned} \textrm{SINR}_{u,{\mathscr{I}}_{u,i}} = \frac{ \rho |{\hat{H}}_{u,{\mathscr{I}}_{u,i}}|^2}{1+ \rho |\tilde{H}_{u,{\mathscr{I}}_{u,i}}|^2}, \end{aligned}$$

where ${\mathscr{I}}_{u,i}$ is the *i*-th entry of the set ${\mathscr{I}}_u$. We remark that ${\hat{H}}_{u,{\mathscr{I}}_{u,i}}$ and $\tilde{H}_{u,{\mathscr{I}}_{u,i}}$ follow a zero-mean complex Gaussian distribution with variances of $1-\varepsilon _{u,{\mathscr{I}}_{u,i}}$ and $\varepsilon _{u,{\mathscr{I}}_{u,i}}$, respectively.

## Performance evaluation

In this section, the PS method is compared with the conventional design for the spectral efficiencies and the bit error rate (BER) performances as well.

### Ergodic capacity

This sub-section investigates the performances of the PS method on the ergodic capacity of the OFDMA system. For a given largest delay spread, considering (14), the ergodic capacity can be calculated by

15 $$\begin{aligned} \eta _{\textrm{ps}} = \frac{2}{L}\sum \limits _{u=1}^{U}\sum \limits _{i=1}^{I_u-I_{u-1}}\mathbb {E}\left\{ \log _2{\left( 1 + \textrm{SINR}_{u,{\mathscr{I}}_{u,i}} \right) } \right\} , \end{aligned}$$

in the units of [bits/sec/Hz], where $\eta _{\textrm{ps}}$ represents the ergodic capacity of the PS method. The factor 2/*L* on the right side of the above equation takes into account the fact that both the *l*-th and $(L-l)$-th data subcarriers are allocated to the same user because data subcarriers of the UE are inserted at both sides of the pilot subcarrier.

Substituting Eq. (9) into Eq. (15), the ergodic capacity of the PS method is obtained by

16 $$\begin{aligned} \eta _{\textrm{ps}} = \frac{2}{L}\sum \limits _{u=1}^{U}\sum \limits _{i=1}^{I_u-I_{u-1}}\mathbb {E}\left\{ \log _2{\left( 1+ \frac{ \rho |{\hat{H}}_{u,{\mathscr{I}}_{u,i}}|^2}{1+ \rho |\tilde{H}_{u,{\mathscr{I}}_{u,i}}|^2} \right) }\right\} , \end{aligned}$$

where *U* is the total number of UEs, ${\mathscr{I}}_u$ is the alloted subcarriers to UE_u_, out of *L* pilot spacing, and ${\mathscr{I}}_{u,i}$, is the *i*-th entry of ${\mathscr{I}}_u$.

For its conventional counterpart^31,32^, each UE is independently distributed with $L-1$ data subcarriers. the ergodic data rate can be calculated by

17 $$\begin{aligned} \eta _{\textrm{con}} = \frac{1}{LU}\sum \limits _{u=1}^{U} \sum \limits _{j=1}^{L-1}\mathbb {E}\left\{ \log _2{\left( 1+ \frac{ \rho |{\hat{H}}_{u,j}|^2}{1+ \rho |\tilde{H}_{u,j}|^2}\right) }\right\} . \end{aligned}$$

### Simulation results

To demonstrate the effectiveness of the PS method, we examine an OFDMA system operating within a bandwidth of 20 MHz, where the individual subcarrier bandwidth is 15 kHz. For simulation setup, it is assumed that the data of eight UEs, $U=8$, is transmitted, with the delay spread values of [3100 ns, 1650 ns]^33^, i.e., ${\bar{\tau }}_1 = 3100$ ns and ${\bar{\tau }}_8= 1650$ ns. In PS method, to design the sub-block coherent bandwidth, the delay spread ${\bar{\tau }}_u$ of the shortest delay spread UE is considered as standard for the calculation of coherence bandwidth. As an example, we use $(L-1)$ data subcarriers and divide them equally between *U* number of UEs, i.e., $I = (L-1)/2(U)$. In this way, each UE has the same number of *I* data subcarriers. In PS method, the data subcarriers are added at both sides of the pilot tone, so *I* is half of the total data subcarriers of a single UE; the factor 2 in Eq. (16) complete the data subcarriers of each UE.

**Figure 5:**
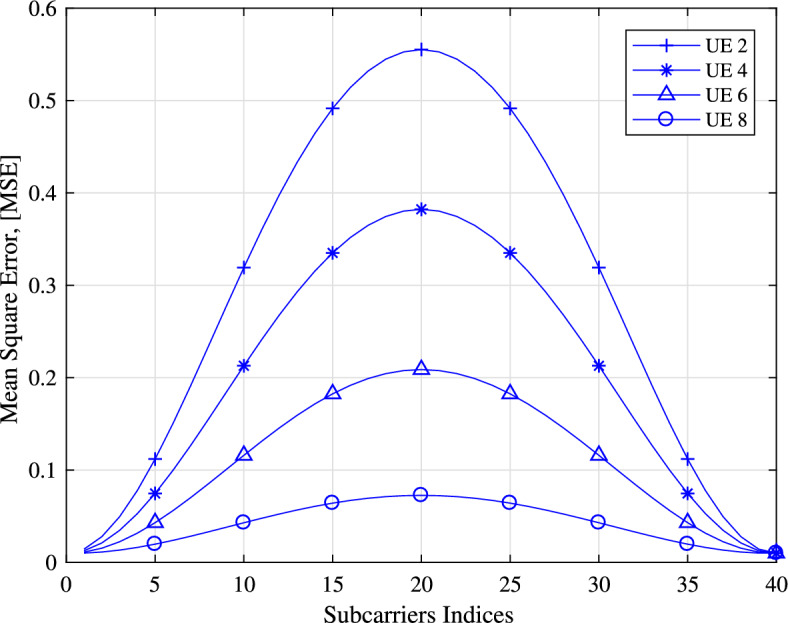
Mean square error plotted for UE 2, UE 4, UE 6, UE 8.

We initially plot the simulation results for the MSE utilized in the channel estimation, derived in Eq. (13) for various values of the indices of the subcarriers in Fig. 5, for UE 2, UE 4, UE 6 and UE 8. The interval of pilot tones *L* equals 40. The estimated MSEs at the *l*-th data subcarrier and the $(L-l)$-th data subcarrier are the same. Meanwhile, the data subcarriers in closer proximity to the pilot tone have smaller estimated MSEs. Furthermore, the MSE decreases with the increase of the UE index, which indicates a more accurate channel estimation for the UEs with smaller delays spread. This is because the CCB increases as the delay spread decreases, causing a larger correlation among the subcarriers. These phenomena validate the feasibility of our PS method, that is, loading symbols of UEs with smaller CCBs into the subcarriers closer to the pilot tones.

**Figure 6:**
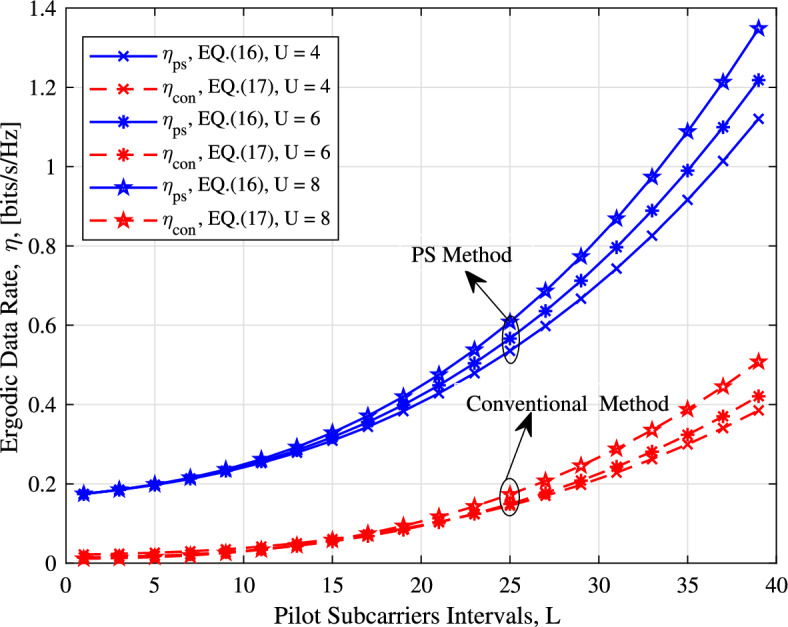
The ergodic data rate of the PS method and its conventional counterpart.

The ergodic data rate of the PS method and its conventional counterpart for $U = 4, \ 6, \ 8$ UEs is plotted in Fig. 6. The signal-to-noise ratio $\rho$ is set to 20 dB. We observe from Fig. 6, that the PS method achieves higher spectral efficiency than its conventional counterpart. Furthermore, the performance gap broadens as the interval of the pilot tones increases. In Fig. 6, with the increase in the number of UEs, the spectral efficiency increases in our model. The primary reason for this is that the difference in spectral efficiency between our PS and the traditional counterpart depends on the ratio of data subcarriers to the total number of subcarriers in the subblock. As the number of UEs increases, the conventional design experiences an increase in pilot retransmissions. However, in the PS design, the existing pilot is shared among the UEs. Therefore, no extra pilot tones are inserted in the OFDMA subblocks.

**Figure 7:**
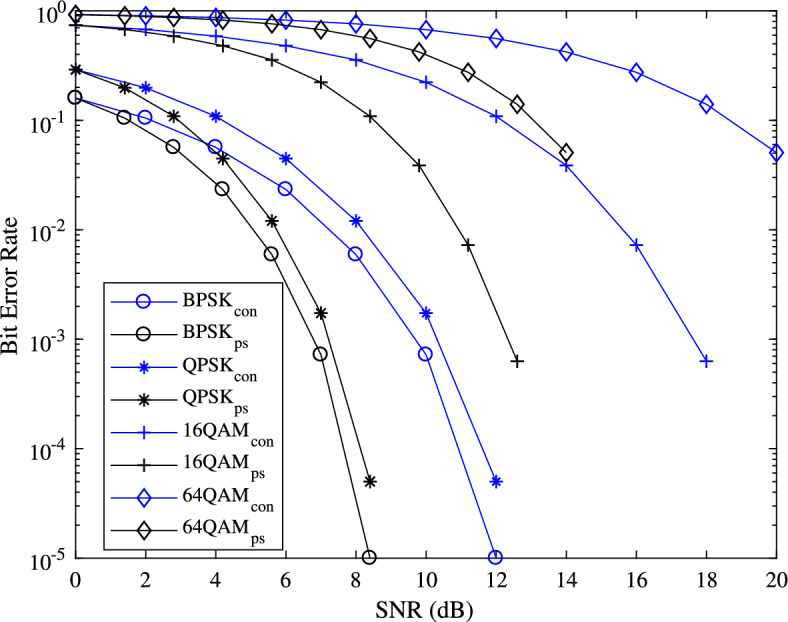
BER performance of UE 1, in the conventional method and PS method.

Figure 7, displays the comparison of the bit error rate (BER) performance of UE 1 between the PS method and conventional method, using the Hard decision method with BPSK, QPSK, 16QAM, and 64QAM modulation. This comparison demonstrates that the PS method outperforms the conventional method in terms of BER. The enhancement in BER is attributed to the insertion of data for UE 1 closer to the pilot in the PS method, while in the conventional method, the data for UE 1 is positioned randomly from the pilot subcarrier.

**Figure 8:**
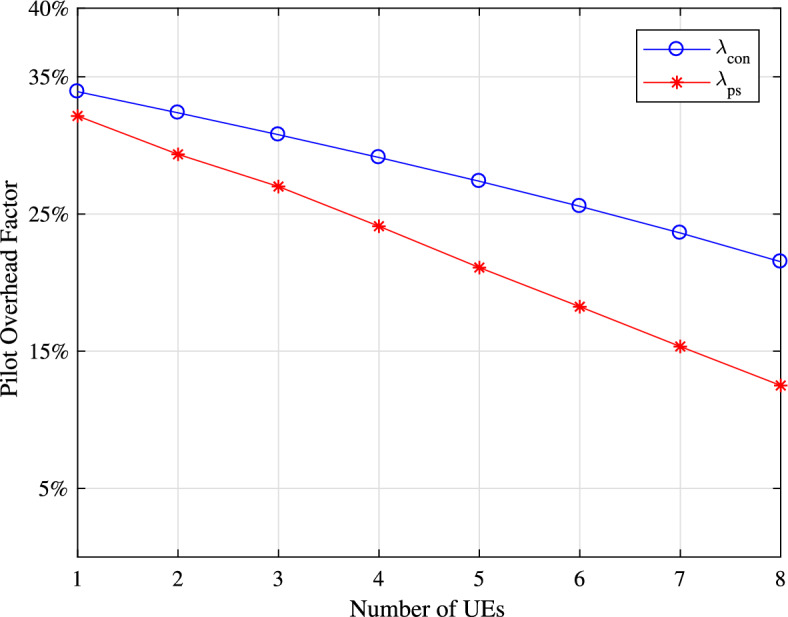
Pilot overhead of PS method and its conventional counterpart.

To clarify the contribution of our PS method more clearly, we computed the pilot overhead for the PS method as

18 $$\begin{aligned} {\uplambda _{ps} }= \left( \frac{P}{L_U}\right) , \end{aligned}$$

where *P* denotes the number of pilot tones generally $P=1$ for a single subblock, $L_U$ stands for the total number of subcarriers’ of shortest delay spread UE and its conventional counterpart,

19 $$\begin{aligned} {\uplambda }_{\textrm{con}} =\left( \frac{UP}{\sum _{u=1}^{U}(L_u) }\right) , \end{aligned}$$

where $L_u$ stands for the number of subcarriers of UE*u* and and $u = 1,2,\ldots , U$. In Eq. (19), as *u* increases, the number of pilot spacings also increases, so we utilize aggregation to compare ${\uplambda }_{\textrm{con}}$ with the PS method ${\uplambda _{ps} }$. Figure 8 illustrates the pilot overhead factor plotted against the number of UEs for $P=1$. As the number of UEs increases, the pilot overhead diminishes notably in the PS method due to the absence of additional inserted pilots. Conversely, in the conventional system, the reduction is marginal because extra pilot tones are inserted. Specifically, the PS method exhibits nearly a $10\%$ enhancement in bandwidth efficiency when $U = 8$.

## Conclusion

This paper presents a novel PS method tailored for orthogonal frequency division multiple access (OFDMA) downlink transmissions. The method strategically allocates data across diverse subcarriers, aiming to enhance bandwidth utilization compared to traditional designs. Theoretical underpinnings are established using the CCB, providing a theoretical framework for evaluation. Through simulation-based assessments, the method’s performance is gauged, highlighting its superiority in spectral efficiency over conventional designs. The results emphasize the PS method’s efficacy in maximizing spectral efficiency, representing a significant stride in downlink transmission techniques for OFDMA systems. This innovation not only underscores improved spectral efficiency but also signifies a pivotal advancement in addressing the critical aspect of bandwidth usage in the dynamic realm of wireless communication technologies.

## Data Availability

The data will made available on a reasonable request to the corresponding author (jiaobl@pku.edu.cn (BJ)).
